# Molecular Connections of Aging and Cancer

**DOI:** 10.14336/AD.2017.0822

**Published:** 2017-10-01

**Authors:** William C. Cho

**Affiliations:** Department of Clinical Oncology, Queen Elizabeth Hospital, Hong Kong

Thanks to advances in health care, more effective therapeutic interventions, hygiene control and food availability, we have an improved human life expectancy in this era. Consequently, aging has become a significant risk factor for several diseases including cardiovascular disease, cancer, neurological disorders, diabetes, and obesity. According to the World Health Organization, life expectancy is now exceeding 80 years in most developed countries. The process of becoming older is a complex scenario influenced by the interaction of various genetic and environmental factors. While aging represents the single most significant risk factor for cancer development, based on the US National Cancer Institute’s Surveillance Epidemiology and End Results (SEER) Database, 43% of men and 38% of women will develop cancer over the course of their lives (1). Almost two-thirds of all new cancer diagnoses are made in persons over the age of 65 years, thus aging is making a larger proportion of people more susceptible to cancer (2).

Aging is an inevitable time-dependent decline in physiological organ function and is a major player in cancer development. It is now clear that aging and cancer development either share or diverge in several disease mechanisms. Such mechanisms include the role of genomic instability, telomere attrition, epigenetic changes, loss of proteostasis, decreased nutrient sensing and altered metabolism. Current research on aging is mostly based on genetic studies in relatively short-lived organisms and animals. The biological basis of human aging is much more complex and thus animal models cannot fully capture the process of cancer development in the aging human being.

Though cancer and aging are both complicated processes with various cellular activities e.g., proliferation and metabolism, the underlying mechanism linking both processes are the time dependent accumulation of cellular damage (3). At the cellular level, aging can cause cellular senescence at which single cells within an organism have ceased dividing. Since age-associated decreases in gene expression could contribute to a progressive decline in cellular function, understanding the mechanisms that determine the aging transcriptome provides a potential target to extend healthy cellular lifespan (4). In this sense, aging and cancer are tightly interconnected and many of the same strategies and drugs may be used to target both, while in other cases antagonistic pleiotropy can come into effect where the inhibition of one can be the activation of the other (5). Indeed, several anti-aging interventions are being actively studied e.g., epigenetic modulators, stem cell therapies, activation of telomerase, autophagy inducers, senolytic therapeutics and plasma membrane redox system activators (6). In fact, some natural products or nutraceuticals have been shown to elicit anti-aging and anticancer effects. The regulation of microRNA expression is a key target of natural products, which have been shown to prevent aging, cancer, diabetes, cardiovascular and other diseases (7,8).

Cellular reprogramming is another mechanism of aging and cancer. One of the views revolving around cellular reprogramming is epigenetic plasticity being a fundamental element of a tissue’s capacity to undergo successful repair, aging degeneration or malignant transformation. Epigenetic plasticity not only provides a stochastic insight into the current deterministic genetic paradigm for most chronic diseases but may also increase our understanding of the connections between physiological aging and cancer (9).

Another interesting hallmark between aging and cancer is inflammation, which also suggests possible molecular targets that may be exploited to modify their actions. For example, aspirin and NSAIDs were suggested to decrease cancer incidence due to their anti-inflammatory effect. On the other hand, immunotherapy may also modulate age-associated cancer development by improving senescent cell clearance (10).

Here, Zinger et al. review different cell autonomous and non-cell autonomous mechanisms that link aging and cancer. They explore the inflammatory perspective, which provides plausible explanations for the similarities between aging and cancer, as well as the contradictions between these two biological processes (11).

From a metabolic perspective, aging and cancer have dysregulated metabolism in their progression, e.g., upregulation of glycolysis and down-modulation of oxidative phosphorylation. Indeed, it is possible that the long-term causative effect of aging could be linked through metabolic control, thus metabolic interventions may be a strategy to promote longevity and alleviate age-related disorders including cancer (12).

Cancers are caused by gene mutations that may be inherited, induced by environmental factors, or result from DNA replication errors (13). However, the stepwise gene mutation theory fails to explain why large animals with more cells do not have a greater cancer incidence than humans (Peto’s paradox). The resurgent studies on the Warburg effect may explain Peto’s paradox and expound the connections of metabolism and cancer. In this special issue, Tidwell et al. discuss these connections and how age-related changes in metabolism are strongly linked to cancer development, which is further affected by lifestyle choices that modulate the risk of aging and cancer through epigenetic control (14).

It is known that ovarian cancer (OC) risk at menopause is significantly modified by parity records during prior fertile life. Urzua et al. reported that they profiled a panel of circulating cytokines in multiparous versus virgin C57BL/6 female mice at peri-estropausal age and investigated how cytokine levels were modulated by intraperitoneal tumor induction in a syngeneic immunocompetent OC mouse model. They found that serum CCL2, IL-10, IL-5, IL-4, TNF-α, IL1-β and IL-12p70 levels increased with age irrespective of parity status, but were specifically reduced following OC tumor induction only in multiparous mice. They concluded that previous parity history counteracts aging-associated systemic inflammation possibly by reducing the immunosuppression that typically allows tumor spread. According to the authors, this is the first report describing a long-term effect of pregnancy on age-associated chronic inflammation relevant to OC in an animal model. Further studies seem warranted (15).

In fact, OC is the third most common and the first cause of death from gynecologic cancer. In the U.S., 22,280 new cases are projected in 2016, 44% of which occur in women over the age of 65 (1). However, excess hazard ratios for death at 1-year and 5-years was higher for old compared to young patients (16). This may be due to older women who are undertreated, receive less chemotherapy or a combination of surgery and chemotherapy, even though this is considered the optimal treatment modality. Thus, while mortality and the incidence of OC increase with a patient’s age, the elderly patients themselves express a strong wish to receive radical and curative treatment (17). This may be mainly due to the lack of evidence and physician’s confidence in the management of elderly women with OC. Thus, Tortorella et al. review the management of older women with OC, considering geriatric features tied to this population. The reason for poorer prognosis in older women is still unclear, such an outcome may be due to a variety of factors (18).

The worldwide surge in the incidence of skin cancer during the last two decades has reached “epidemic” proportions, resulting from long, lifetime sun exposure in an increasingly aging population (19).

Skin cancer is heterogeneous in nature, comprising cutaneous melanoma and non-melanoma skin cancers (NMSC), which predominantly affect elderly patients. Compared to younger patients, the clinical presentations of melanoma are diverse in elderly patients. Therefore, clinical management should be tailored for these two groups of patients according to their age-related variations. It was noted that many were frequently undertreated because of limited access to advanced surgical and medical treatments. Current clinical practice guidelines for melanoma and NMSC only partially address the geriatric aspects of cancer care, including frailty, limited life-expectancy, geriatric comorbidities and treatment compliance. The review by Garcovich et al. addresses the recent evidence and problem of skin cancer in the elderly population as well as age-related variations in its clinical management, highlighting the potential role of a geriatric approach in optimizing dermato-oncological care (20).

The National Institute on Aging classify elderly persons into young-old (aged 65-75), old (aged 76-85) and oldest-old (older than 85 years), but there is no general agreement on the age at which a person becomes old. In reality, chronic health conditions are commonplace in older populations. Aging is a global health concern and cancer is an age-related disease. However, limited information is available concerning the epidemiology of age-related cancer and the contribution of cancer to the mortality of the 85 years and older group (21). The oldest-old is the fastest growing age group in the U.S. and some other nations. Due to chronic disease, the oldest-old have the highest population levels of disability that require long-term care. Over time, more older people will survive to even more advanced ages, thus more attention needs to be paid to the elderly population. The global cancer burden is growing with the increase in population aging. It is my hope that this special issue will garner increased attention and interest from the community to pursue more studies on age-related cancer mechanisms.
